# Anti-Metastatic and Anti-Tumor Growth Effects of *Origanum* *majorana* on Highly Metastatic Human Breast Cancer Cells: Inhibition of NFκB Signaling and Reduction of Nitric Oxide Production

**DOI:** 10.1371/journal.pone.0068808

**Published:** 2013-07-10

**Authors:** Yusra Al Dhaheri, Samir Attoub, Kholoud Arafat, Synan AbuQamar, Jean Viallet, Alaaeldin Saleh, Hala Al Agha, Ali Eid, Rabah Iratni

**Affiliations:** 1 Department of Biology, College of Science, United Arab Emirates University, Alabama, Ain, United Arab Emirates; 2 Department of Pharmacology and Therapeutics, Faculty of Medicine and Health Sciences, United Arab Emirates University, Alabama, Ain, United Arab Emirates; 3 Institut National de la Sante et de la recherche Medicale U823, Université Joseph Fourier, Grenoble, France; 4 Department of Biological and Environmental Sciences, College of Arts and Sciences, Qatar University, Doha, Qatar; Institute of Molecular and Cell Biology, Biopolis, United States of America

## Abstract

**Background:**

We have recently reported that *Origanum* *majorana* exhibits anticancer activity by promoting cell cycle arrest and apoptosis of the metastatic MDA-MB-231 breast cancer cell line. Here, we extended our study by investigating the effect of *O* *. majorana* on the migration, invasion and tumor growth of these cells.

**Results:**

We demonstrate that non-cytotoxic concentrations of *O* *. majorana* significantly inhibited the migration and invasion of the MDA-MB-231 cells as shown by wound-healing and matrigel invasion assays. We also show that *O* *. majorana* induce homotypic aggregation of MDA-MB-231 associated with an upregulation of E-cadherin protein and promoter activity. Furthermore, we show that *O* *. majorana* decrease the adhesion of MDA-MB-231 to HUVECs and inhibits transendothelial migration of MDA-MB-231 through TNF-α-activated HUVECs. Gelatin zymography assay shows that *O* *. majorana* suppresses the activities of matrix metalloproteinase-2 and -9 (MMP-2 and MMP-9). ELISA, RT-PCR and Western blot results revealed that *O* *. majorana* decreases the expression of MMP-2, MMP-9, urokinase plasminogen activator receptor (uPAR), ICAM-1 and VEGF. Further investigation revealed that *O* *. majorana* suppresses the phosphorylation of IκB, downregulates the nuclear level of NFκB and reduces Nitric Oxide (NO) production in MDA-MB-231 cells. Most importantly, by using chick embryo tumor growth assay, we also show that *O* *. majorana* promotes inhibition of tumor growth and metastasis *in vivo*.

**Conclusion:**

Our findings identify *Origanum* *majorana* as a promising chemopreventive and therapeutic candidate that modulate breast cancer growth and metastasis.

## Introduction

Breast cancer is the leading cause of cancer-related deaths in women worldwide. Approximately one-third of all women with breast cancer develops metastasis and ultimately dies as a result of the effects of the disease [1,2]. Cancer metastasis starts in the primary tumor site when cancer cells start to invade and degrade the basement membrane and the extracellular matrix (ECM) (invasion) into the vascular or lymphatic circulation and then survive in the circulation. Loss of cell adhesion, induces the disassembly of cancer cells from the primary tumor, disseminating them to distant sites through blood vessels and lymphatics, and eventually leave the circulation to establish metastasis in distant organs [3,4]. E-cadherin, a cell–cell adhesion molecule, plays a major role in the establishment and maintenance of normal tissue architecture. It is expressed predominantly on the surface of normal epithelial cells. For cancer cells to become metastatic, they must decrease E-cadherin expression and break these cell-cell adhesions associated and induction of cell mobility triggering a transition from tumorigenic (epithelial) to migratory/invasive (mesenchymal) phenotype ending in tumor metastasis. Hence, the expression level of the epithelial cadherin (E-cadherin) has become an important indicator for these transitions. Therefore, searching for agents that could enhance E-cadherin expression may be attractive therapeutic target for repressing the metastatic potential of cancer cells [5,6].

Adhering of tumor cells to endothelial cells is an essential step during cancer progression and metastasis. Several adhesive molecules, such as intracellular adhesion molecule-1 (ICAM-1), have been identified as being responsible for the endothelial adhesion of cancer cells [7]. While ICAM-1 was found to be expressed at a low basal level in many cell type including epithelial and endothelial cells [8], its expression as well as soluble serum ICAM-1 were found to be high in metastatic breast cancer patients [8]. Therefore, agents that repress ICAM-1 expression in breast cancer cells and subsequently blocks the interaction between cancer and endothelial cells might be an important therapeutic target for repressing the metastatic potential of cancer cells.

Angiogenesis is a complex multistep process involving soluble factors, adhesion molecules, proteases and cytokines. The process of tumor angiogenesis starts when tumor cells themselves secrete and activate angiogenic factors, thereby activating proteolytic enzymes. At this time, endothelial cells concurrently proliferate, migrate, and differentiate. Vascular endothelial growth factor (VEGF) is the most prominent mediator in tumor angiogenesis that is markedly induced in breast cancer [9]. Up-regulation of VEGF expression has been reported in a variety of malignant human cancers including breast, colon, lung cancers. An in situ hybridization study of human breast samples showed high VEGF expression in the tumor cells but not the normal duct epithelium [10]. Hence, VEGF might be a good target in the treatment of breast cancer patients.

Degradation of the extracellular matrix (ECM) surrounding the primary tumor is an essential step in cancer cells invasion. This degradation is important for tissue remodeling and induction of angiogenesis, and is mainly mediated by specific proteolytic enzymes systems mainly matrix metalloproteinases (MMPs) and urokinase plasminogen activator (uPA). Among all MMPs, upregulation of MMP-2 and MMP-9 was shown to be associated with breast cancer metastasis and poor clinical outcome [11]. Northern Blot analysis revealed that the level of MMP-2 and MMP-9 mRNA transcript was higher in breast cancer tissue compared to normal breast tissue [12]. In addition, higher MMP-9 protein concentration was detected in breast cancer tissue when compared to normal breast tissue [13]. Similarly, higher protease activity for MMP-2 and MMP-9 was detected by zymography in tumor tissue compared to normal tissue [14].

MMPs are directly activated by the serine protease plasmin, which is activated from its proenzyme form (plasminogen) by the serine protease urokinase-type plasminogen activator (uPA) upon binding to cell surface receptor (uPAR). Overexpression of uPA has been found in many tumor types and is correlated with poor prognosis. Moreover, binding of uPA to uPAR also induces signal transduction that allows enhanced cell migration [11,15–17]. Therefore, regulating the expression of ECM degradation enzymes are considered as a therapeutic target for breast cancer.

Nuclear factor κB (NFκB), a transcription factor, is a key player in cancer metastasis [3]. It has been shown that several genes involved in tumor metastasis are directly regulated by NFκB. The frequent over-expression of NFκB in tumor cells suggests that selected tumor cells may acquire metastatic activity by aberrant expression of metastasis relevant genes during their progression. In fact, studies from different groups showed that MMP-2 and MMP-9 [1,16], uPA [18,19], uPAR [20] and ICAM-1 [21] are downstream targets of NFκB signaling pathway.

Phytochemicals have accumulated increasing importance with regards to their ability to decrease various tumors growth, such as those in the breast. In regards to breast cancer, increasing data have shown that these phytochemicals modulate multiple pathways used by cancer cells in the processes of cell proliferation, survival, angiogenesis, invasion, and metastasis [9]. Disruption of one or more of these pathways, therefore, provides an effective avenue for therapeutic intervention of breast cancer. In this regards, several phytochemicals have been reported to be effective in inhibiting the invasive potential of many metastatic cancer cell lines. Examples of such promising phytochemicals include: [6]-gingerol [17], isothiocyanates from broccoli and watercress [11], tea catechins [22], genistein, apigenin [23], Ganoderma lucidum [24], and ganoderic acid from the *G* *. lucidum* [25].

*Origanum* *majorana* , commonly known as marjoram, is spread worldwide. It is utilized as a spice and flavoring agent, and in traditional medicine as well for the treatment of chest infection, cough, sore throat, rheumatic pain, nervous disorders, stomach disorders, cardiovascular diseases, and skin care. There is increasing evidence that *O* *. majorana* possesses extensive range of biological activity, including antioxidant, antimicrobial, anti-inflammatory and hepatoprotective activities [26–30]. Recently, we have shown that *Origanum* *majorana* suppresses the growth of the triple negative MDA-MB-231 breast cancer cells by causing cell cycle arrest and apoptosis [31]. However, its effect against tumor invasion and metastasis is largely unknown. In this study, we have extended our investigation by testing the ability of *O* *. majorana* to inhibit migration, invasion and metastasis of the MDA-MB-231 cells.

## Materials and Methods

### Preparation of the *Origanum* *majorana* ethanolic extract (OME)

*Origanum* *marjorana* ethanolic extract (OME) was prepared as previously described [31].

#### Cell culture and reagents

Human breast cancer cells MDA-MB-231 and MDA-MB-231-GFP were maintained in DMEM (Hyclone, Cramlington, UK). MDA-MB-23-GFP used in this study were previously described [32]. All media were complemented with 10% fetal bovine serum (FBS) (Hyclone, Cramlington, UK), 100U/ml penicillin/streptomycin (Hyclone, Cramlington, UK). HUVECs (Invitrogen, Carlsbad, CA, USA) were maintained in MEM 199 supplemented with 20% FBS, penicillin/streptomycin, 2 mM L-glutamine, 5U/ml heparin and 50 µg/ml endothelial cell growth supplements (BD Biosciences, Bedfrord, MA, USA). Antibodies to NF-κB p65 (3034), pIκB-α (ser32) (2859) were obtained from Cell Signaling (Cell Signaling Technology, Inc., Danvers, MA, USA). Antibodies to ICAM-1 (sc-107), β-actin (C4, sc-47778), goat anti-mouse IgG-HRP (sc-2005) and goat anti-rabbit IgG-HRP (sc-2004) were obtained from Santa Cruz Biotechnology, Inc. Antibody to E-cadherin (ab15148) and uPAR (ab 103791) were obtained from Abcam and AlexaFluor 488 goat anti-rabbit IgG (H+L) (A11008) was obtained from invitrogen.

#### Wound healing migration assay

Wound healing assay was performed as previously described [33]. Briefly, MDA-MB-231 cells were grown in six-well tissue culture dishes until confluence. A scrape was made through the confluent monolayer with a sterile plastic pipette tip of 1mm diameter. Afterwards, the dishes were washed twice with PBS and incubated at 37°C in fresh DMEM complemented with 10% fetal bovine serum in the presence or absence of the indicated concentrations of OME. At the bottom side of each dish, three arbitrary places were marked where the width of the wound was measured with an inverted microscope (objective x 10). Wound closure was expressed as the average ± SEM of the difference between the measurements at time zero and the 4-10 h time period considered.

#### Migration Chamber Assay

5 x 10^4^ cells were seeded in the upper chamber, HTS multiwell insert system (BD Biosciences, Franklin Lakes, NJ, USA), in serum-free medium, with or without OME and migration assay was carried out according to the manufacturer’s instruction. Serum-containing medium was added to the lower chamber to act as a chemotactic attractant. After 6 hours of incubation, cells were washed with PBS, fixed with formaldehyde. Cells were then stained with 1% crystal violet for 10 minutes. After washing with PBS, cells from at least 5 different random fields were counted under a microscope.

#### Cellular viability

Cells were seeded in triplicate in 96-well plates at a density of 5,000 cells / well. After 24h of culture, cells treated with or without OME and incubated for the indicated time period. Cell viability was determined using a CellTiter-Glo Luminescent Cell Viability assay (Promega Corporation, Madison, WI, USA), based on quantification of ATP, which signals the presence of metabolically active cells. Luminescent signal was measured using Berthold FB12 Luminometer. Data were presented as proportional viability (%) by comparing the treated group with the untreated cells, the viability of which is assumed to be 100%.

#### Aggregation Assay

Growing cells were detached using 2mM EDTA in calcium magnesium-free PBS (CMF-PBS). A cell suspension of 1 x 10^6^ cells/ml was aliquoted into microcentrifuge tubes, washed with PBS, resuspended in 1m culture media and incubated with or without OME on a rocker for 1 h at 37°C. Cells were then fixed with 1% formaldehyde and pictures taken under an inverted microscope (Olympus IX71). Aggregation was calculated as previously reported [34] using the following equation: % aggregation=(1-Nt/Nc)x100, where Nt and Nc represent the number of single cells in treated or untreated groups, respectively.

#### Adhesion Assay

Adhesion of MDA-MB-231 to HUVECs was performed as previously described [35] with minor modification. Briefly, cell culture plates were coated with collagen and HUVECs grown to confluent monolayers. TNF-α (25ng/ml) was added for 6 hours to stimulate HUVECs prior to the addition of MDA-MB-231 cells. MDA-MB-231 transfected with *Renilla* luciferase were resuspended in HBSS containing 1% BSA at a concentration of 2x10^5^ cells/ml. 150 µl of this cell suspension was added to the upper chamber containing confluent HUVECs in the absence or presence of OME. After 1 hour, unattached cells were removed by gently washing the plates three times with PBS. Adherent cells were lysed using a luciferase lysis buffer (Promega, Madison, WI, USA) and light units measured according to the manufacturer instructions.

#### Matrigel invasion assays

Invasion assay was performed as previously described [33]. The invasiveness of the MDA-MB-231 cell treated with the indicated concentrations of OME was tested using BD Matrigel Invasion Chamber (8-µm pore size; BD Biosciences, Bedfrord, MA, USA) according to manufacturer’s instructions. Briefly, MDA-MB-231 (1 x 10^5^) cells placed in 0.5 mL of media containing vehicle or the indicated concentrations of OME were seeded into the upper chambers of the system; the bottom wells in the system were filled with DMEM complemented with 10% foetal bovine serum as a chemo-attractant and then incubated at 37°C for 24h. Non-penetrating cells were removed from the upper surface of the filter with a cotton swab. Cells that have migrated through the matrigel were fixed with 4% formaldehyde, stained with DAPI and counted in 6 random fields under a microscope. For quantification, the assay was done in duplicates and repeated three times.

#### Measurement of matrix metalloproteinases by ELISA

Cells were seeded in 6-well plates in the absence of vehicle or OME for 24 hours. The conditioned medium was collected and the levels of secreted MMP-2 and MMP-9 were determined using immunoassay kits (Invitrogen, Camarillo, CA, USA) according to the manufacturer’s protocol. Experiments were repeated three times and the average of three means is represented ± SEM.

#### Gelatin zymography

Gelatin zymography was performed as previously described [36]. Briefly, MDA-MB-231 (2.5 x 10^6^) cells were incubated in serum-free DMEM for 24 h in the presence of vehicle or OME (150 and 300 µg/mL). The conditioned medium was collected, concentrated and 30 µg of total protein was resolved in 10% polyacrylamide gels containing 0.1% gelatin. After electrophoresis, the gels were washed for 1 h in 2.5% (v/v) Triton X-100 to remove SDS and then incubated overnight at 37 °C in 50 mM Tris-HCl (pH 7.5), 150 mM NaCl, 0.5 mM ZnCl_2_ and 10 mM CaCl_2_ to allow proteolysis of the gelatin substrate. Bands corresponding to activity were visualized by negative staining using 0.5% Coomassie brilliant blue R-250 (Bio-Rad, Hercules, CA, USA). Representative results from two independent experiments are shown. Densitometry was performed using ImageJ software and band density was normalized to the non-specific band staining on the gel.

#### Immunofluorescence staining

immunofluoreacence staining was performed as previously described [31]. MDA-MB-231 cells (3 x10^4^) were grown in complete media on 4 well labtek chamber slide (Nunc, Rochester, NY, USA) for 24 h, then treated with ethanol as control or OME extract for 24 h at the indicated concentrations. Cells were then fixed in 10% formalin solution (4% paraformaldehyde) (Sigma-Aldrich, Saint-Quentin Fallavier, France) for 5 min at RT followed by permeabilization in PBS containing 0.1% Triton X-100 for 5 min at RT. Cells were then washed three times with PBS, blocked with 5% nonfat dry milk in PBS for 30 min at RT and incubated with the primary antibody diluted, at the concentration suggested by the manufacturer, in 1% nonfat dry milk/PBS overnight at 4^°^C. Following overnight incubation, cells were washed three times with PBS and placed for 1 h at RT in the presence of fluorescein-conjugated secondary antibody diluted at 1:200 in 1% nonfat dry milk/PBS. After washing with PBS, cells were mounted in Fluoroschield with DAPI (Sigma-Aldrich, Saint Louis, MO, USA) and examined under Nikon Ti U fluorescence microscope.

#### Quantitative Immunoassay for Human Vascular Endothelial Growth Factor (VEGF)

MDA-MB-231 cells (1.5 x 10^5^) were seeded in 24-well plates. The conditioned medium was collected and the level of VEGF therein measured using a VEGF enzyme-linked immunosorbent assay kit (R&D Systems, Minneapolis, MN, USA) according to the manufacturer’s protocol. Assays were performed in triplicates and three independent experiments were performed. Data are presented as mean values ± SEM**.**

#### Quantification of Nitrate/Nitrite production

The amount of Nitrate/Nitrite production was determined with a colorimetric ELISA kit (Cayman Chemical, Ann Arbor, Michigan, USA), which is based on the Griess reaction, according to the manufacturer’s instructions. The value of nitrate/nitrite presented is the total value measured in the presence of cells minus the value determined from the media alone in the absence of any growing cells.

#### Luciferase activity

MDA-MB-231 cells were seeded in 12-well plates the day before transfection. Cells were then transfected with the E-cadherin luciferase reporter expression plasmid [37] using Lipofectamine 2000 (Life Technologies, Inc. Grand Island, NY, USA) according to the manufacturer’s protocol. Briefly, cells were allowed to recover for 4 hours after transfection in OPTI-MEM (Life Technologies, Inc. Grand Island, NY, USA) which was then replaced with complete medium. Luciferase activity was measured using Dual Luciferase Reporter Assay System (Promega, Madison, WI, USA). *Renilla* luciferase reporter was used as an internal control, to which firefly luciferase values were normalized. Experiments were repeated three times and the average of three means is represented ± SEM.

#### Transendothelial Migration Assay

Transendothelial migration of MDA-MB-231 through HUVEC was performed as previously described [29]. Briefly, transwell filters were coated with collagen and allowed to dry overnight. HUVECs (2 X 10^5^/well) were then seeded onto the rehydrated membrane and allowed to grow until a confluent monolayer is formed. Where mentioned, TNF-α (25ng/ml) was added for 6 hours to stimulate HUVECs. Then, MDAMB 231 cells (1 x 10^6^) were then loaded on top and incubated overnight in the absence or presence of OME. Cells on the upper chamber were removed with a cotton swab, whereas MDA-MB-231 on the bottom were stained and quantified as previously reported [29].

#### RNA extraction and RT-PCR

Total RNA from vehicle- or OME-treated MDA-MB-231 cells were prepared using Trizol reagent as described by the manufacturer (Life Technologies, Inc. Grand Island, NY, USA). RNA expression of MMP-2 and MMP-9 was determined by RT-PCR. RT-PCR was performed using the Qiagen OneStep RT-PCR kit (Qiagen, Hilden, Germany) according to manufacturer’s instruction. Equal amounts of RNA (500 ng) were used as templates in each reaction. The sequences of specific primers were as follows: MMP-2 sense, 5′-TCTCCTGACATTGACCTTGGC -3′, and antisense: 5′-CAAGGTGCTGGCTGAGTAGATC -3′; MMP9 sense, 5′- TTGACAGCGACAAGAAGTGG-3′, and antisense, 5′- CCCTCAGTGAAGCGGTACAT-3′; GAPDH sense, 5′-GGCCTCCAAGGAGTAAGACC -3′, and antisense: 5′- AGGGGTCTACATGGCAACTG-3′. The PCR products were separated by 1.5% agarose gel and visualized by ethidium bromide staining. Representative results from two independent experiments are shown.

#### Nuclear and whole Cell extract and Western Blotting analysis

Cells (1.8 x 10^6^) were seeded in 100mm culture dishes and cultured for 24h before addition of various concentrations of OME extract. For whole cell lysates, after incubation for the indicated times, cells were washed twice with ice-cold PBS, released by scrapping, pelleted and lysed in RIPA buffer supplemented with protease/phosphatase inhibitor cocktail. Following incubation for 30 min on ice, the cell lysate was obtained by centrifugation at 14,000 rpm for 20 min at 4^°^C. Nuclear extract were prepared from vehicle- or OME-treated MDA-MB-231 cells using the NE-PER extraction reagents (Thermo Scientific, Rockford, IL, USA) according to the manufacturer’s instructions. Protein concentration of lysates was determined by BCA protein assay kit (Thermo Scientific, Rockford, IL, USA) and the lysates were adjusted with lysis buffer. Aliquots of 30 µg of total proteins were resolved onto 10-12% SDS-PAGE. Proteins were transferred to nitrocellulose membranes (Thermo Scientific, Rockford, IL, USA) and blocked for 1 h at room temperature with 5% non-fat dry milk in TBST (TBS and 0.05% Tween 20). Incubation with specific primary antibodies was performed in blocking buffer overnight at 4^°^C. Horseradish peroxidise-conjugated anti-IgG was used as secondary antibody. Immunoreactive bands were detected by ECL chemiluminescent substrate (Thermo Scientific, Rockford, IL, USA). Membrane stripping by incubating the membrane in Restore western blot stripping buffer (Thermo Scientific, Rockford, IL, USA) according to the manufacturer’s instructions.

#### Chick embryo tumor growth and metastasis assay

The chick embryo tumor growth assay was performed as previously described [38] with slight modifications. According to the French legislation, no ethical approval is needed for scientific experimentations using oviparous embryos (decree n° 2013-118, February 1, 2013; art. R-214-88). Animal work was done under animal experimentation permit N° 381029 to jean Viallet and animal experimentation permit N° B3851610001 to Institut Albert Bonniot. Briefly, Fertilized White Leghorn eggs (Société Française de Production Agricole, St.-Brieuc, France), were incubated at 38°C with 60% relative humidity for 10 days. At stage E10, the chorioallantoic membrane (CAM) was dropped by drilling a small hole through the eggshell into the air sac and a 1 cm^2^ window was cut in the eggshell above the CAM. Cultured MDA-MB-231-GFP were detached by trypsinization, washed with complete medium and suspended in serum free DMEM. A 50 µl inoculum of 1 X 10^6^ MDA-MB-231-GFP cells was added onto the CAM of each egg (eggs were randomized in 4 groups of 15). One day later, tumors that began to be detectable were treated every second day at E11, E13, E15 and E17 by dropping 100 µl of either OME (300 µg/mL or 450 µg/mL), colchicine (2µM) or 0.02% ethanol (vehicle) in PBS onto the tumor. At E19 the upper portion of the CAM was removed, transferred in PBS and the tumors were then carefully cut away from normal CAM tissue and weighted. In parallel, a 1cm^2^ portion of the lower CAM was collected to evaluate the number of nodules, containing GFP-expressing cells. The fluorescent nodule were visualized *in situ* using whole mounts of tissue fixed in 4% formaldehyde in PBS and flattened between a hollow glass slide and a thick coverslip. In order to number the nodule, a thorough and complete visual scan of the piece of the lower CAM was done using fluorescent microscope. Chick embryos were sacrificed by decapitation.

#### Statistical analysis

Results were expressed as means ± S.E.M. of the number of experiments. A Student’s *t*-test for paired or unpaired values was performed and a *p* value of < 0.05 was considered statistically significant.

## Results

### *Origanum* *majorana* attenuates the migration ability of the MDA-MB-231 breast cancer cells

**Figure 1:**
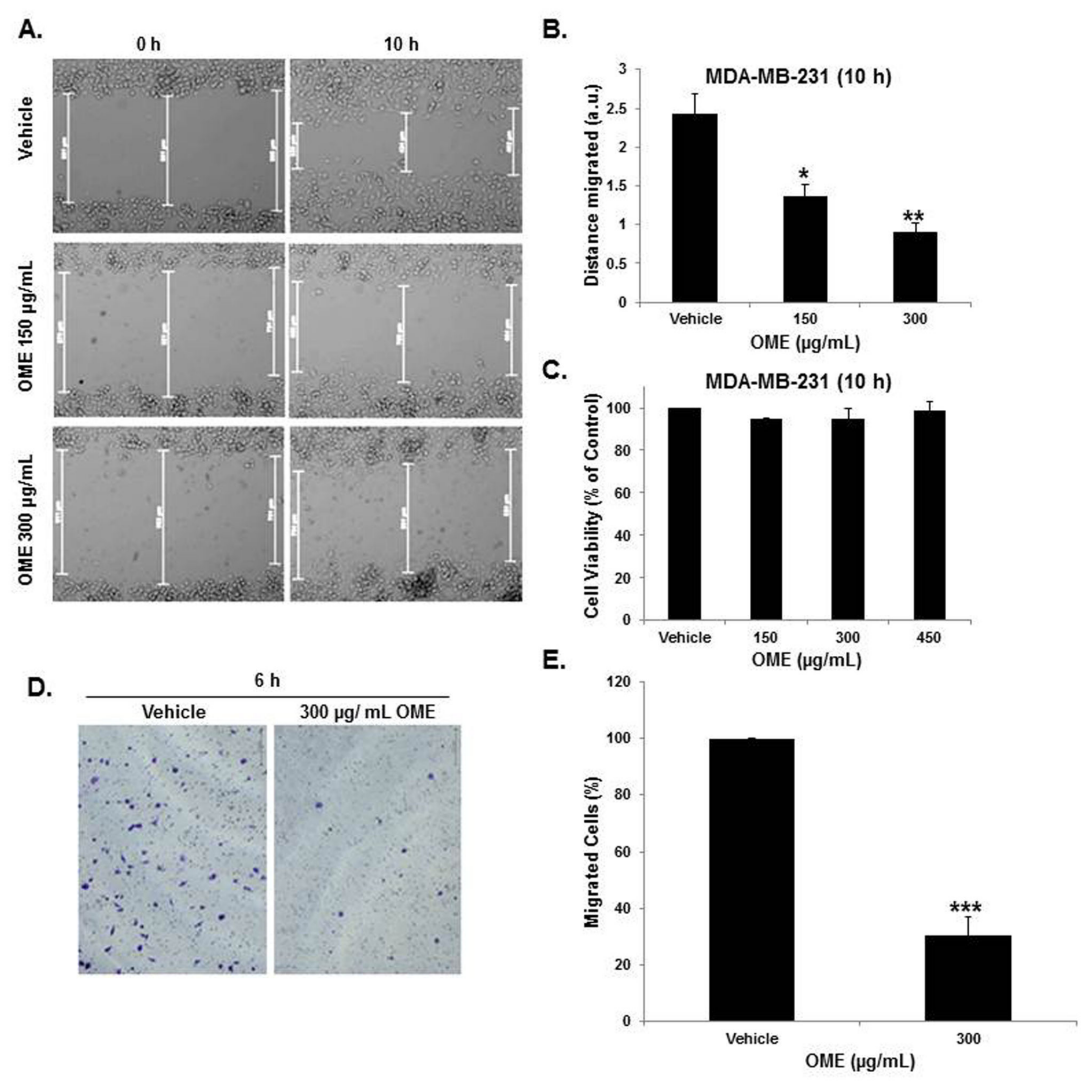
*O* *. majorana* extract inhibits the migration of the MDA-MB-231 human breast cancer cells. (A) Confluent culture of MDA-MB-231 cells were wounded by scratching with a pipette tip and the cells were incubated in DMEM supplemented with 10% fetal bovine serum without and with indicated concentrations of OME. The wound was measured with an inverted microscope (x 100 magnification) and photographed. (B) Wound healing assay showing that OME inhibited the migration of MDA-MB-231 breast cancer cells in dose-dependent manner. Values represent means ± SEM (n=3) of the difference between the measurements at time zero and the 4-10 h time period considered. (C) Cell viability (in percent) determined by using CellTiter-Glo Luminescent Cell Viability assay, after incubation of the exponentially growing cells with or without various concentrations of OME for 10 h. (D) Boyden chamber transwell assay indicating that OME inhibited the migration of MDA-MB-231 cells. (E) Quantification of Migrated MDA-MB-231 cells. Migrating cells from at least 5 different random fields were counted under a microscope. Values represent means ± SEM, n=3. Student’s t test was performed to determine the significance (*p < 0.05, **p < 0.005 and ***p < 0.0005).

We have recently reported that OME induces cell cycle arrest and apoptosis in MDA-MB-231 cells [31]. Because cell migration plays a crucial role in tumour metastasis, we sought to investigate whether OME affects the migration behaviour of MDA-MB-231 cells, we first measured the migration ability of these cells by using wound-healing migration assay. For this purpose, were performed the test with concentrations of OME and periods of treatment that were previously shown to be non-cytotoxic to the MDA-MB-231 [31]. As shown in Figure 1A and 1B, OME treatment significantly inhibited wound healing cellular migration of MDA-MB-231 cells in a concentration-dependent manner. The ability of OME to inhibit the migration of MDA-MB-231 cells was also measured by using the Boyden chamber transwell assay. For this purpose, MDA-MB-231 cells were seeded in the upper wells with or without 300 µg/mL OME. As it is shown in Figure 1D and 1E, the number of OME-treated cells that has migrated to the lower chamber, after 6 h treatment, was significantly reduced by approximately three fold compared to the number of vehicle-treated cells. Cell viability of the OME-treated cells at the concentrations and time tested was not affected (Figure 1C) thus confirming that the inhibitory effect on the MDA-MB-231 cells motility was not due to a cytotoxic effect of OME. Taken together our data confirm the inhibitory effect OME on the migration potential of MDA-MB-231 cells.

### *Origanum* *majorana* promotes cell-cell aggregation and induces E-cadherin upregulation in MDA-MB-231 cells

**Figure 2:**
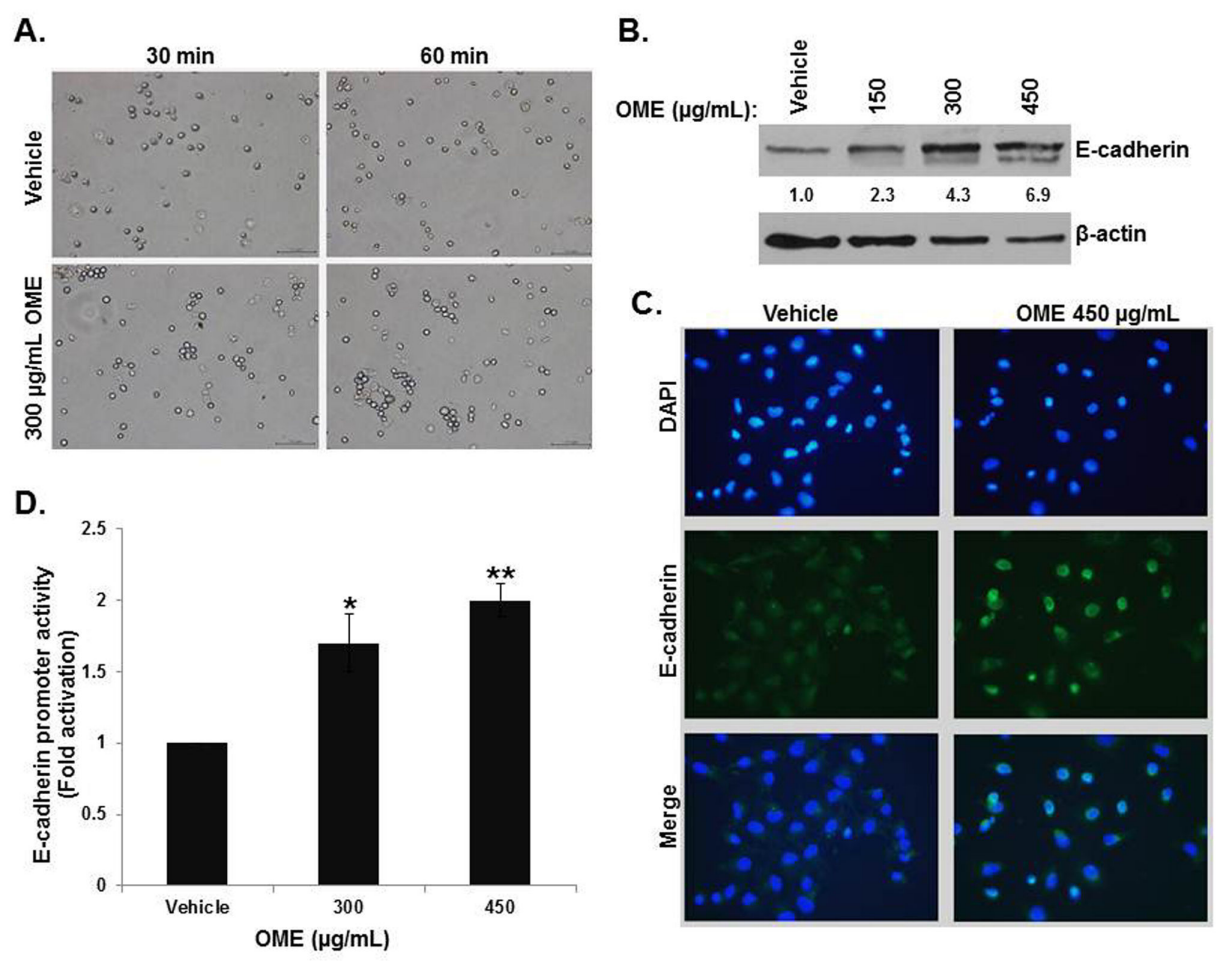
*O* *. majorana* promotes cell-cell aggregation and upregulates E-cadherin expression. (A) MDA-MB-231 cells were incubated without or with 300 µg/mL OME and subjected to cell aggregation as described in Materials and Methods. Aggregated cells were photographed under phase-contrast microscope (x 100 magnification). (B) Western blotting analysis of E-cadherin expression in OME-treated MD-MB-231 cells. Cells were treated with vehicle or increasing concentrations of OME (150, 300 and 450 µg/mL) for 24 h, then whole cell extracts were subjected to Western blot analysis for E-cadherin and β-actin (loading control) proteins. (C) MDA-MB-231 cells were treated with 450 µg/mL OME for 24 h, fixed permeabilized, and then processed for immunofluorescence using antibody against E-cadherin protein. DAPI was used as a nuclear stain. (D) Following transfection with a luciferase reporter gene containing E-cadherin promoter, MDA-MB-231 cells were treated for 24 h with or without OME, lysed, and luciferase activity was measured. The luciferase activities were normalized to the levels of internal Renilla luciferase activity. Experiments were performed in triplicate and repeated three times. Values represent means ± SEM, n=3. Student’s t test was performed to determine the significance (*p < 0.05 and **p < 0.005).

Lost capacity for homotypic adherence is also associated with cancer cell metastasis. To determine whether OME would affect the cell-cell adherence behaviour of MDA-MB 231, cell aggregation assay was performed on vehicle and OME-treated cells. We found that OME significantly increased the ability of MDA-MB-231 cells to form cell aggregates visible as early as 30 min post-treatment (Figure 2A). It is known that loss of E-cadherin expression promotes tumor progression and metastasis while its overexpression prevents invasion of tumor cells. We therefore sought to examine the expression of E-cadherin in MDA-MB-231 cells in response to OME exposure. We first examined the protein level of E-cadherin in MDA-MB-231 cells treated without and with OME. As it is shown in Figure 2B, OME induced a significant increase of E-cadherin protein in a concentration-dependent manner. This increase in E-cadherin expression was further confirmed by immunofluorescence staining (Figure 2C). The expression of E-cadherin was mostly detected at the cells junctions.

Next we determined whether the effect of OME on E-cadherin expression was mediated through a transcriptional regulation. Toward this, transcription activity was measured in cells transfected with a luciferase reporter gene containing E-cadherin promoter and treated without or with OME. As shown in Figure 2D, OME induced a concentration-dependent increase in the luciferase activity, thus indicating that OME positively regulates the expression of E-cadherin at the transcriptional level. Taken together, our data clearly shows that E-cadherin is upregulated by OME and further suggests that the expression of this protein could account for the inhibition of cellular migration and invasion.

### *Origanum* *majorana* inhibits the invasive capacity of MDA-MB-231 cells

**Figure 3:**
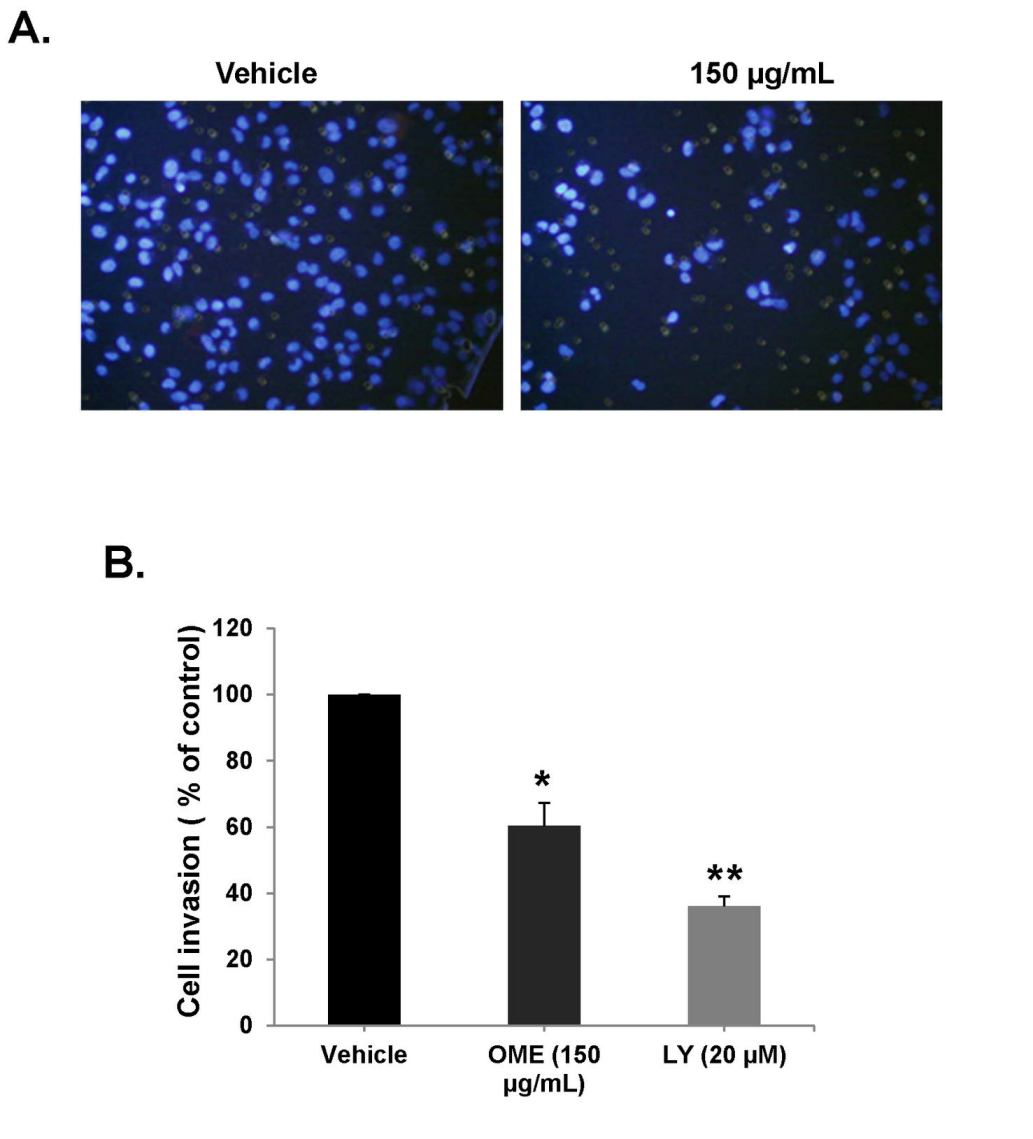
*O* *. majorana* inhibits the invasive activity of MDA-MB-231 cells. (A) MDA-MB-231 cells were incubated for 24 h with or without OME (150 µg/mL) and LY294002 (20 µM). Cells that invaded into the matrigel were scored as described in Materials and Methods. (B) Quantification of invaded MDA-MB-231 into the matrigel. Values represent means ± SEM, n=3. Student’s t test was performed to determine the significance (*p < 0.05 and **p < 0.005).

Next, we examined the invasive potential of MDA-MB-231 cells in the Matrigel-coated Boyden chamber in the absence or presence of 150 µg/mL OME. The number of OME-treated cells that has passed through the Matrigel coated membrane was markedly reduced by 55% (Figure 3, A and B), indicating that OME can inhibit the invasiveness ability of the MDA-MB-231 cells.

### *Origanum* *majorana* suppresses the expression and the activity of MMP-2 and MMP-9 and downregulates uPAR in MDA-MB-231 cells

**Figure 4:**
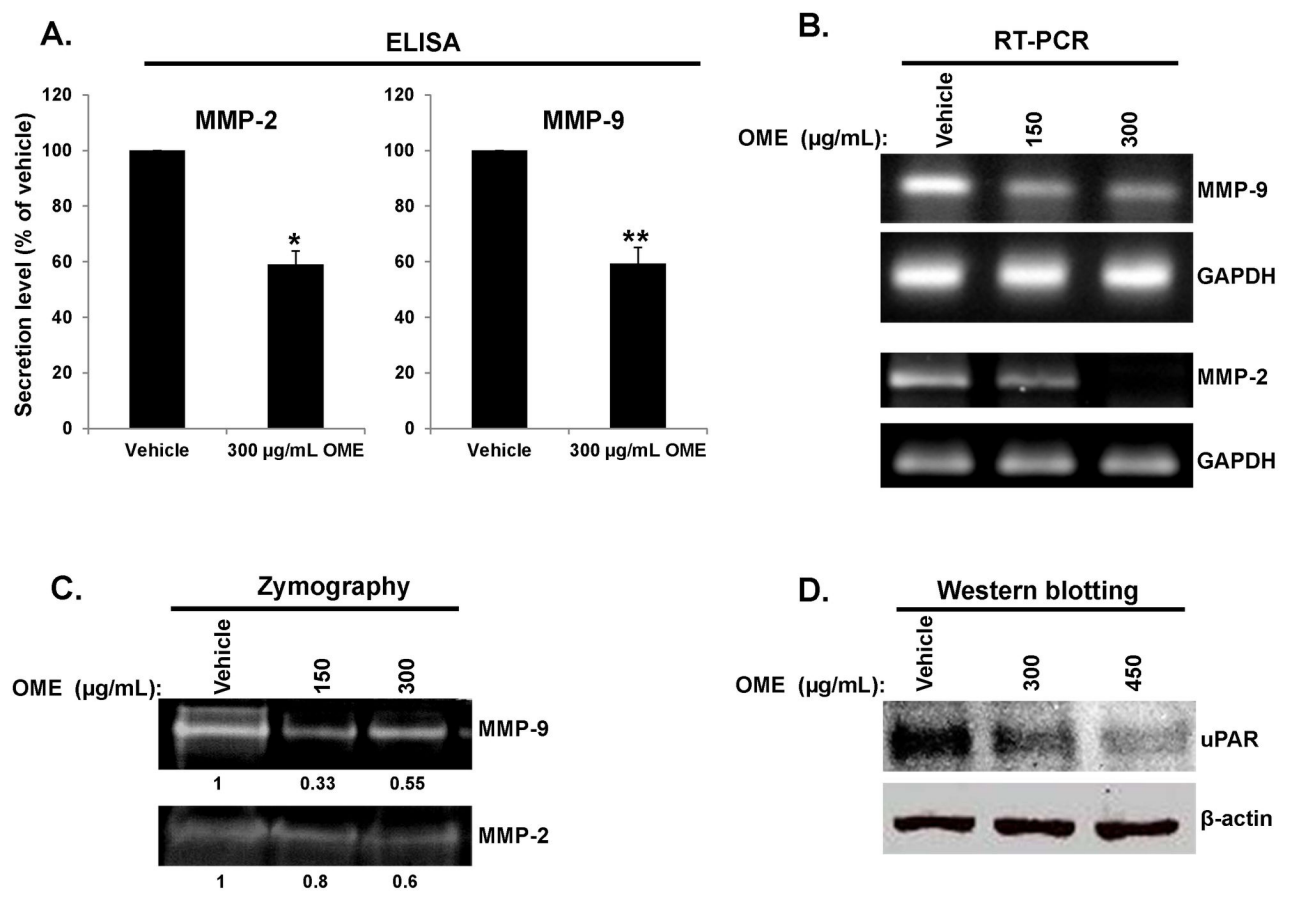
The effect of *O* *. majorana* on MMP-2, MMP-9 and uPAR in MDA-MB-231 cells. (A) Effects of OME on the secretions of MMP-2 and MMP-9 in the collected conditioned medium of OME-treated MDA-MB-231 cells. The levels of secreted MMP-2 and MMP-9 were determined using immunoassay kits as described in Materials and Methods. Experiments were repeated three times and the average of three means is represented ± SEM. Student’s t test was performed to determine the significance (*p < 0.05 and **p < 0.005). (B) Effects of OME one the expression of MMP-2 and MMP-9 mRNA. MDA-MB-231 cells were treated with OME (150 and 300 µg/mL) for 24 h and then subjected to RT-PCR to analyze the mRNA level of MMP-2 and MMP-9. GAPDH was used as an internal control. (C) Activities of MMP-2 and MMP-9 in OME-treated MDA-MB-231 cells. Cells were treated with 150 and 300 µg/mL OME for 24 h and then subjected to gelatin zymography, as described in Materials and Methods, to analyze the activities of MMP-2 and MMP-9. (D) Western blotting analysis of uPAR expression in OME-treated MD-MB-231 cells. Cells were treated with vehicle or increasing concentrations of OME for 24 h, then whole cell extracts were subjected to Western blot analysis for uPAR and β-actin (loading control) proteins.

Matrix metalloproteinases (MMP) -2 and -9, among other MMPs, are known to play an important role in breast cancer cell invasion and metastasis. To test whether *O* *. majorana* inhibits breast cancer cell invasion by affecting the expression of MMP-2 and MMP-9, we decided to examine the protein expression level of MMP-2 and MMP-9 in the conditioned medium using OME-treated MDA-MB-231 cells. The protein level of MMP-2 and MMP-9 (Figure 4A) was found to be significantly reduced in response to OME treatment. RT-PCR analysis also revealed that the MMP-2 and MMP-9 mRNA level was reduced in MDA-MB-231 cells upon treatment with OME (Figure 4B) indicating that OME can inhibit the transcription of MMP-2 and MMP-9 genes in these cells.

Next, we decided to examine the effect of OME on the activity of MMP-2 and MMP-9. MDA-MB-231 cells were treated with 150 and 300 µg/mL OME for 24 h in serum free DMEM, the media was collected, concentrated and tested for MMP2 and MMP-9 activity by gelatin zymography. As it is shown in Figure 4C, MMP-2 and MMP-9 activities were significantly reduced in response to OME treatment. Altogether, our results showed that OME significantly inhibits both, the expression and the activities of MMP-2 and MMP-9.

The urokinase plasminogen activator (uPA) and its receptor (uPAR) were also shown to play a crucial role in breast cancer cell invasion and metastasis. Therefore, we examined whether OME also alters the expression of uPAR. Western blot analysis clearly shows that the expression level of uPAR was markedly decreased in OME-treated MDA-MB-231 cells (Figure 4D).

### *Origanum* *majorana* decreases adhesion of MBA-MB-231 to HUVEC and downregulates the expression of ICAM-1 in the breast cancer cells

**Figure 5:**
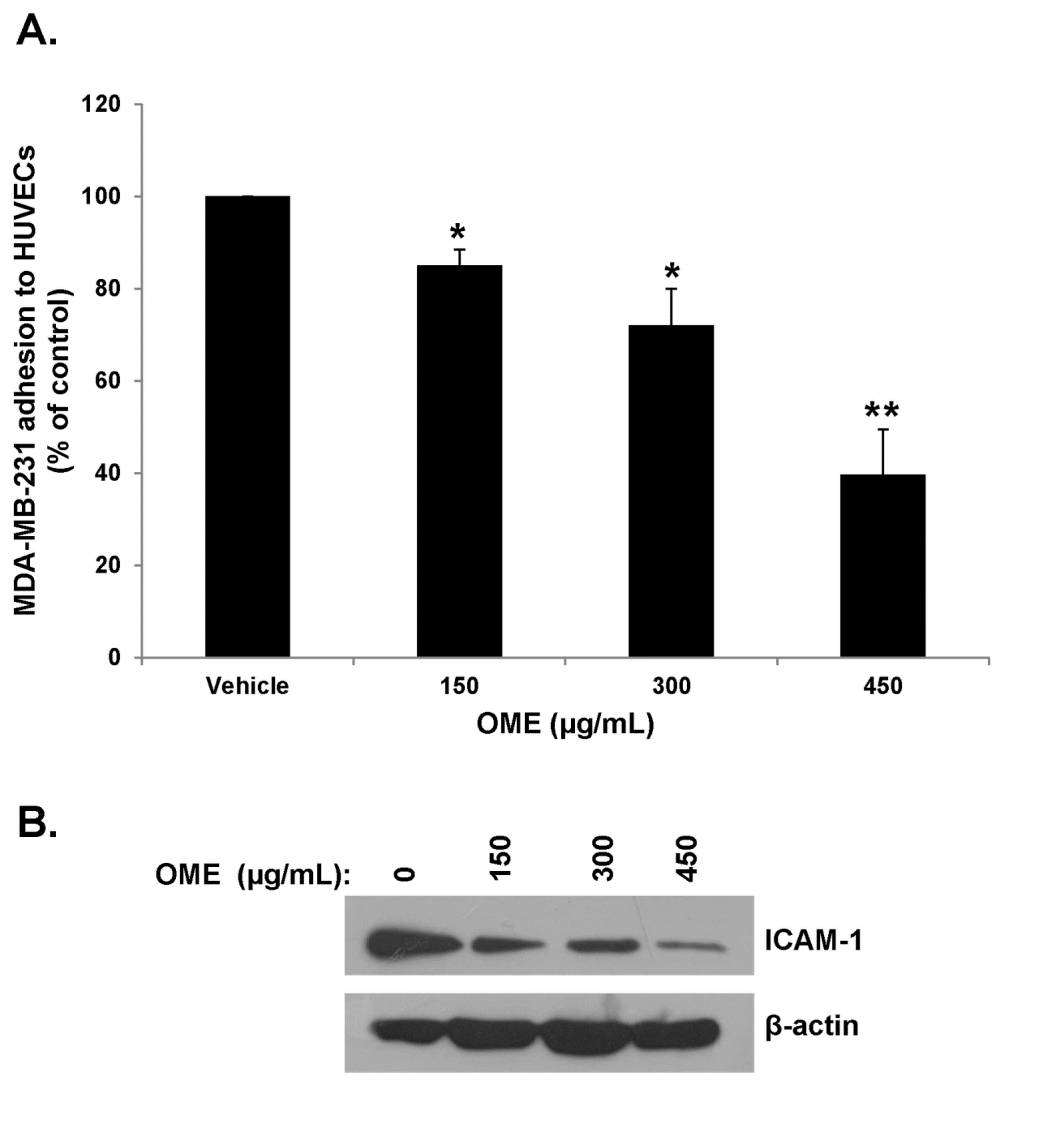
Inhibition of adhesion of MDA-MB-231 cells to HUVEC by *O* *. majorana* . (A) MDA-MB-231 cells were treated overnight with vehicle or OME then seeded on top of the confluent TNF-α stimulated HUVECs and allowed to adhere for 60 min as described in Materials and Methods. The adhesion of MDA-MB-231 cell is represented as percentage of cells that have adhered to HUVECs. (B) Western blotting analysis of ICAM-1 expression in OME-treated MD-MB-231 cells. Cells were treated with vehicle or increasing concentration of OME (150, 300 and 450 µg/mL) for 24 h, then whole cell extracts were subjected to Western blot analysis for ICAM-1 and β-actin (loading control) proteins.

The attachment of tumor cells to endothelial blood vessels is also crucial event in the process of metastasis. The effect of OME on MDA-MB-231 cell adhesion to TNF-α stimulated HUVECs was then investigated by co-incubating both cell types for 1 h with or without various concentrations of OME. Figure 5A shows that OME significantly inhibited the adhesion of MDA-MB-231 cells to HUVECs in concentration-dependent manner.

Because the intercellular adhesion molecule (ICAM)-1, has been shown to play an important role in the adhesion of cancer cells to endothelial cells and therefore in metastasis, we sought to examine whether OME affects the expression of this adhesion molecule in MDA-MB-231 cells. Toward this aim, cells were treated with various concentrations of OME and the protein level of the ICAM-1 was determined by Western blotting. As it is shown in Figure 5B, the level of ICAM-1 protein decreased in concentration-dependent manner in OME-treated MDA-MB-231 cells. Taken together, our results suggest that OME exerts an inhibitory effect on the adhesion of MDA-MB-231 cells to HUVEC and that this effect is associated with a downregulation of ICAM-1protein.

### *Origanum* *majorana* inhibits transendothelial migration of MDA-MB-231 through TNF-α-stimulated HUVECs

**Figure 6:**
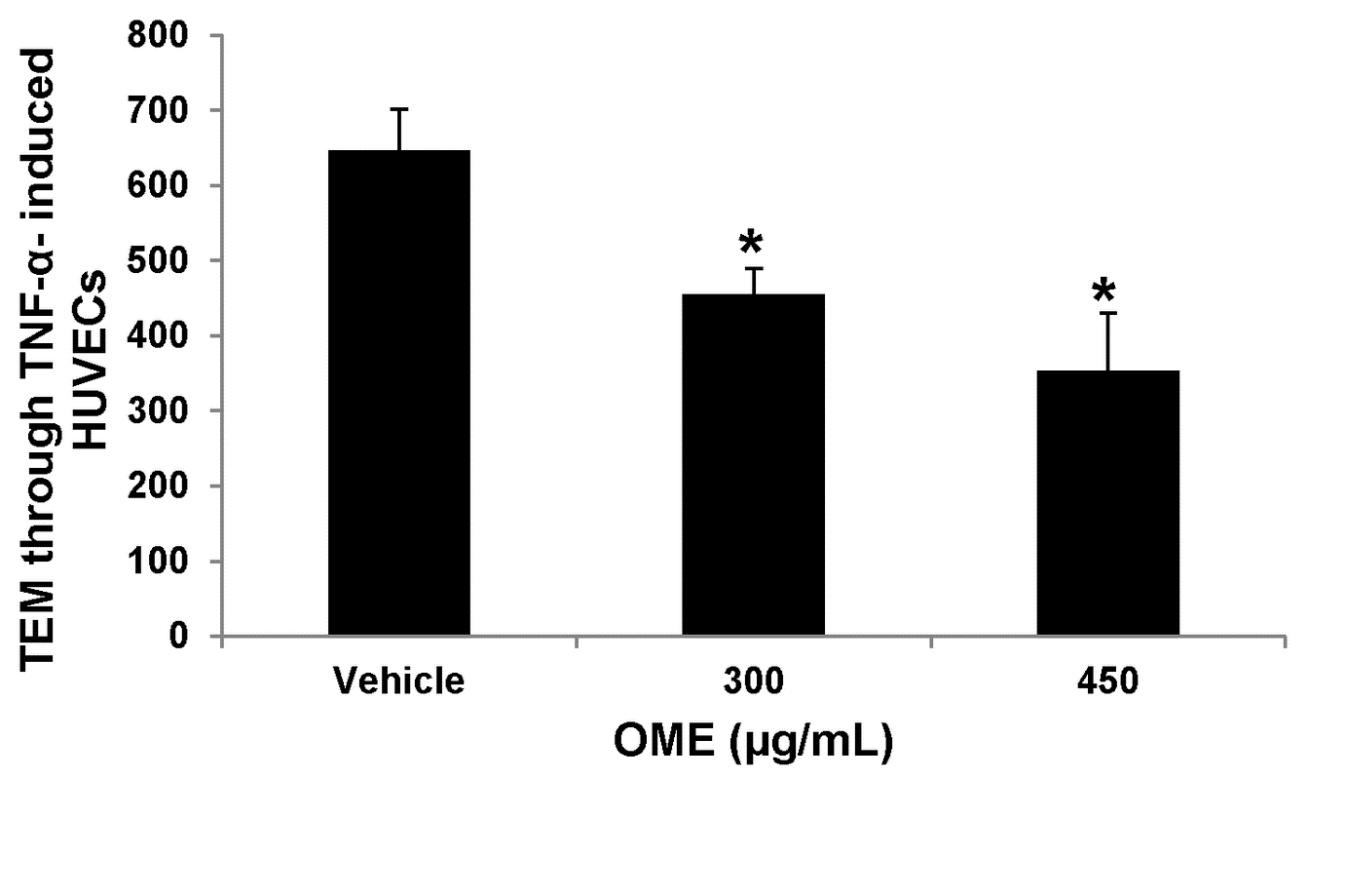
*O* *. majorana* inhibits the migration of MDA-MB-231 cells across monolayer of TNF-α activated HUVECs. Confluent monolayers of HUVECs were pre-incubated with TNF-α (25 ng/mL) for 6 h, and transendothelial migration was evaluated after overnight incubation at 37°C. Values represent means ± SEM, n=3. Student’s t test was performed to determine the significance (*p < 0.05).

Since the migration of tumor cells through the vascular endothelium is another crucial event in the metastasis process, we used the transendothelial migration assay to investigate the effect of OME on the ability of MDA-MB-231 cells to migrate across a monolayer of endothelial (HUVEC) cells. As seen in Figure 6, the transendothelial migration of OME treatment through the monolayer of HUVEC was significantly reduced by the treatment with OME in a dose-dependent manner.

### *Origanum* *majorana* suppresses VEGF production in HUVECs and MDA-MB-231 cells

**Figure 7:**
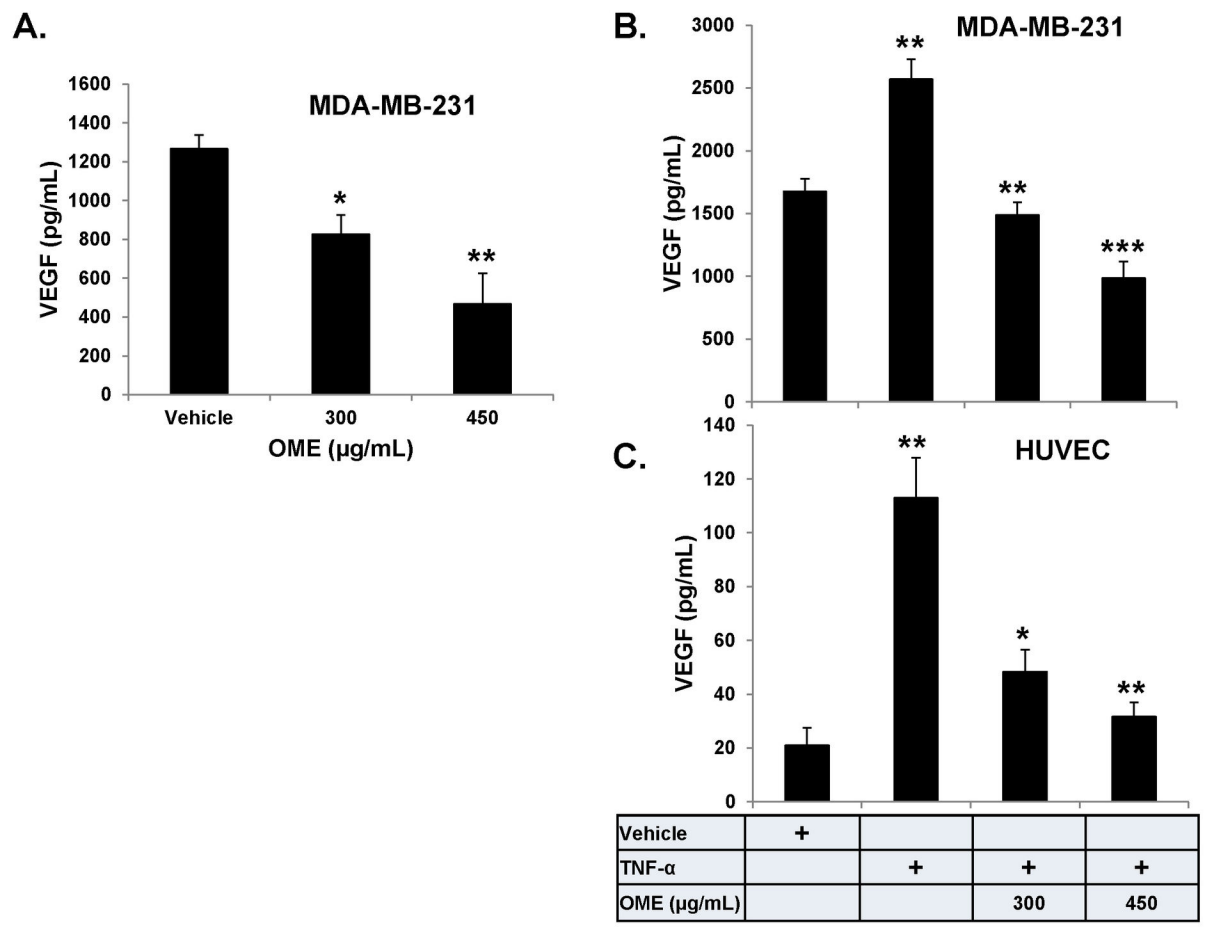
Reduction of VEGF secretion in *O* *. majorana-treated* HUVECs and MDA-MB-231 cells. (A) Quantification of basal level of VEGF secretion in conditioned medium from vehicle or OME-treated MDA-MB-231 cells. Cells were treated with vehicle or the indicated concentrations of OME for 24 h and then the secreted VEGF in the conditioned medium was analyzed by ELISA. Reduction of VEGF secretion in TNF-α induced MDA-MB-231 cells (B) and HUVEC (C) cultured in presence of vehicle or indicated concentrations of OME. VEGF secretion was quantified as by ELISA as described above. Data represents means ± SEM of three independent experiments. Student’s t test was performed to determine the significance (*p < 0.05, *p < 0.005 and **p < 0.0005).

Tumor growth and metastasis critically depend on angiogenesis. VEGF, a pro-angiogenic growth factor, has been shown to play an important role in this process. Thus, we investigated the effect of OME on the production of VEGF by both the MDA-MB-231 and endothelial cells (HUVEC) without or with the presence of TNF-α. We first examined the effect of OME of basal expression level of VEGF in MDA-MB-231 cells. Cells we grown with various concentrations of OME and the conditioned medium were collected and the level of VEGF measured by ELISA. Data shown on Figure 7A revealed that treatment with 300 and 450 µg/mL OME for 24 h markedly reduced the secretion of VEGF by MDA-MB-231 cells. VEGF level dropped from 1300 pg/mL in vehicle treated to 800 and 400 pg/mL respectively. To further confirm the inhibition of VEGF production in breast cancer cells, we measured the level of VEGF in MDA-MB-231 cells that were first treated with various concentration of OME and then stimulated with TNF-α. As shown in Figure 7B, the production of VEGF was enhanced in TNF-α - stimulated (2600 pg/mL) compared to vehicle-treated (1500 pg/mL) MDA-MB-231 cells. However, VEGF production was significantly reduced in concentration-dependent manner by OME (Figure 7B). Next, we examined VEGF production in HUVECs. As expected, HUVEC cultured in the absence of TNF-α produced low level of VEGF (20 pg/mL), while in the presence of TNF-α, VEGF production increased to approximately 120 pg/mL (Figure 7C). Exposure of HUVEC to OME also led to a concentration -dependent suppression of VEGF production (Figure 7C).

### *Origanum* *majorana* inhibits the phosphorylation of IκB, downregulates the nuclear level of NFκB

**Figure 8:**
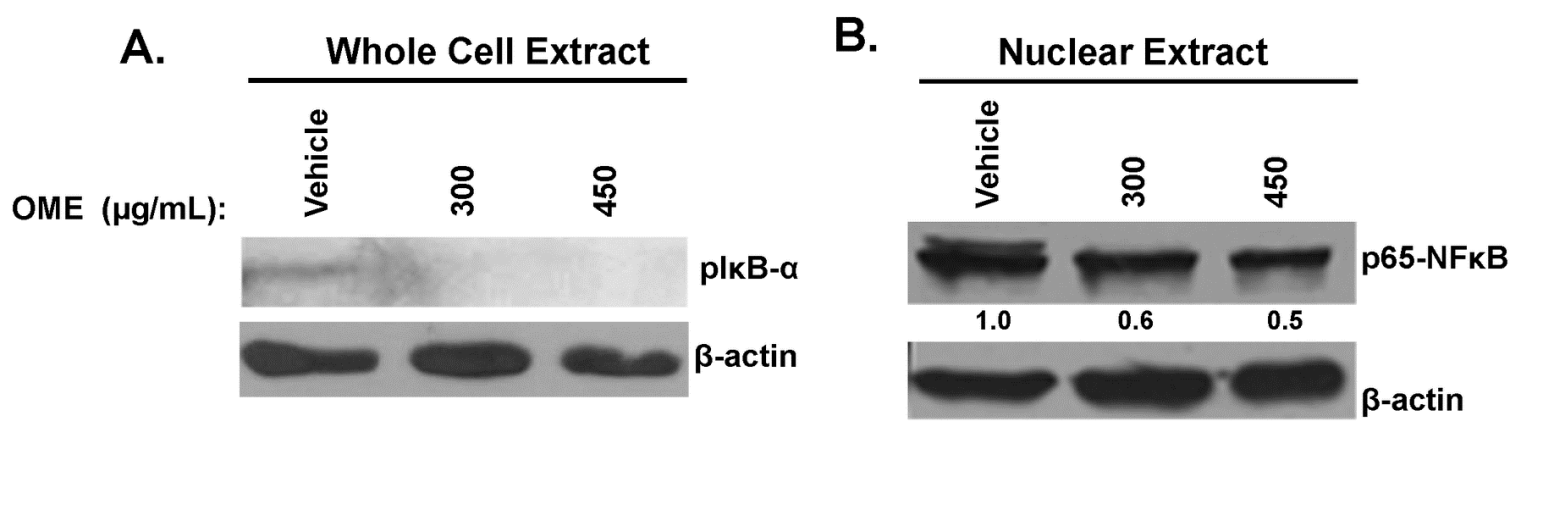
Effect of *O* *. majorana* on NFκB signaling. (A) Western blot analysis of the phosphorylation status of IκBα. MDA-MB-231 cells were treated with vehicle or increasing concentration of OME (300 and 450 µg/mL) for 24 h, then whole-cell extracts were subjected to Western blot analysis for IκBα and β-actin (loading control) proteins. (B) MDA-MB-231 cells were treated with vehicle or indicated concentrations of OME for 24 h and then nuclear extracts were prepared as described in Materials and Methods and subjected to Western blot analysis for p65 (NFκB) and β-actin (loading control) proteins.

The NFκB signaling pathway is known to regulate the expression of various genes involved in tumor cells invasion. To investigate the effect of OME on the activation of NFκB signaling pathway, we first examined, by Western blotting, the phosphorylation status of IκB in OME-treated MDA-MB-231 cells. We found that OME drastically inhibited the phosphorylation of IκB (Figure 8A). Moreover, we found that OME remarkably reduced the level of nuclear NFκB (Figure 8B). Taken together, our data clearly indicates the OME exert its effects at least partly through an inhibition of the NFκB signaling pathway.

### *Origanum* *majorana* reduces Nitric Oxide (NO) production in MDA-MB-231 cells

**Figure 9:**
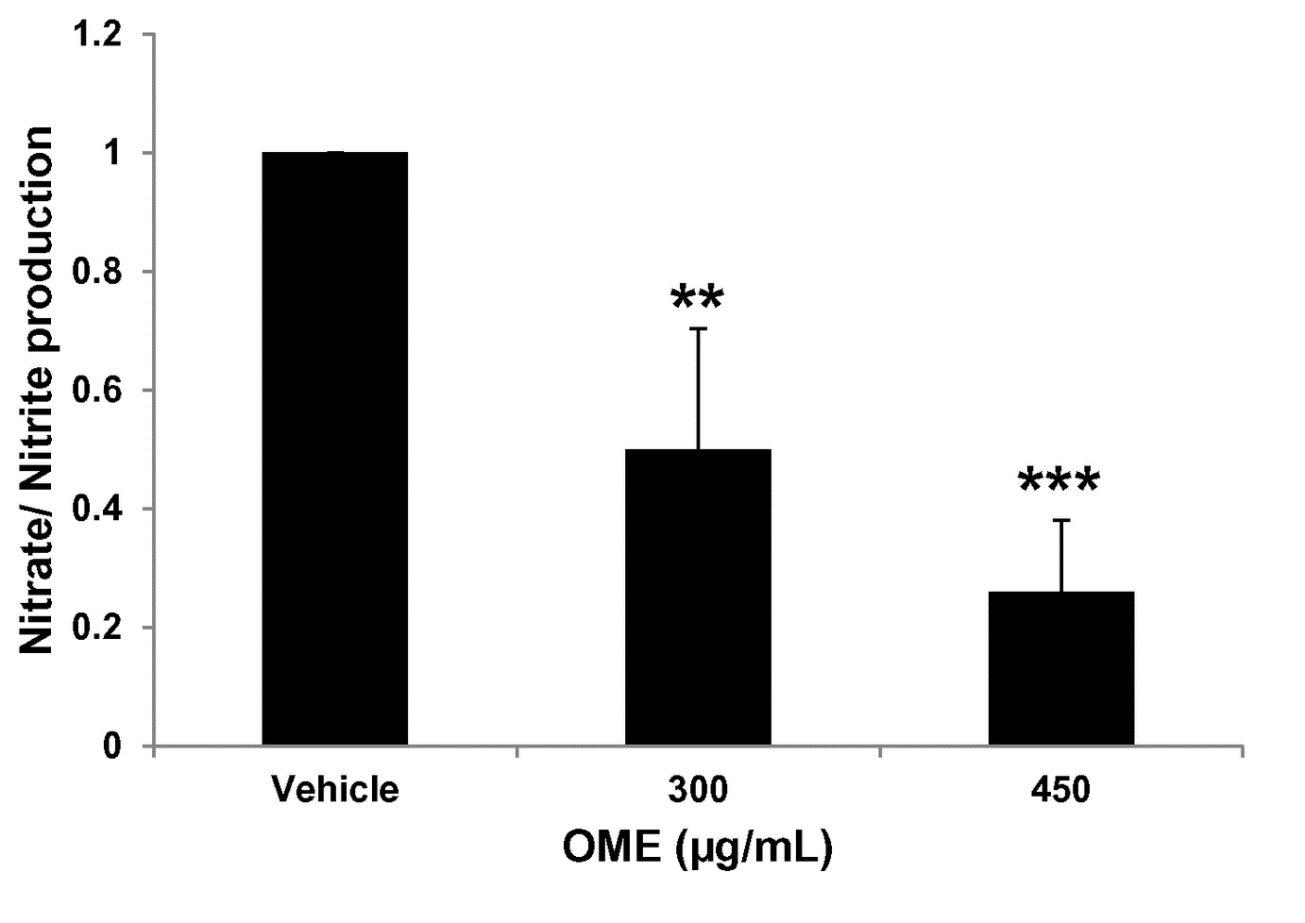
Reduction of Nitric Oxide (NO) production in *O* *. majorana-treated* MDA-MB-231 cells. Quantification of NO levels in OM-treated MDA-MB-231 cells. Cells (1.5 X 10^5^) were treated with vehicle or indicated concentrations of OME for 24 h and Nitrate/Nitrite production was quantified as described in Materials and Methods. Data represents means ± SEM of three independent experiments carried out in triplicate. Student’s t test was performed to determine the significance (*p < 0.05, *p < 0.005 and ***p < 0.0005).

Nitric oxide (NO) signaling has also been shown to promote breast tumor growth and metastasis by altering the expression of genes implicated in cellular migration, invasion and angiogenesis [39–41]. To test whether OME could affect the level of NO, the amount of Nitrate/Nitrite production was determined by ELISA in vehicle and OME-treated MDA-MB-231 cells. Results shown in Figure 9 clearly show that OME decreased NO production in a concentration-dependent manner in MDA-MB-231 cells, thus suggesting that OME could also exert its anti-metastatic effect by modulating the level of NO in breast cancer cells.

### *Origanum* *majorana* inhibits tumor growth and metastasis in chick embryo tumor growth and metastasis assay

**Figure 10:**
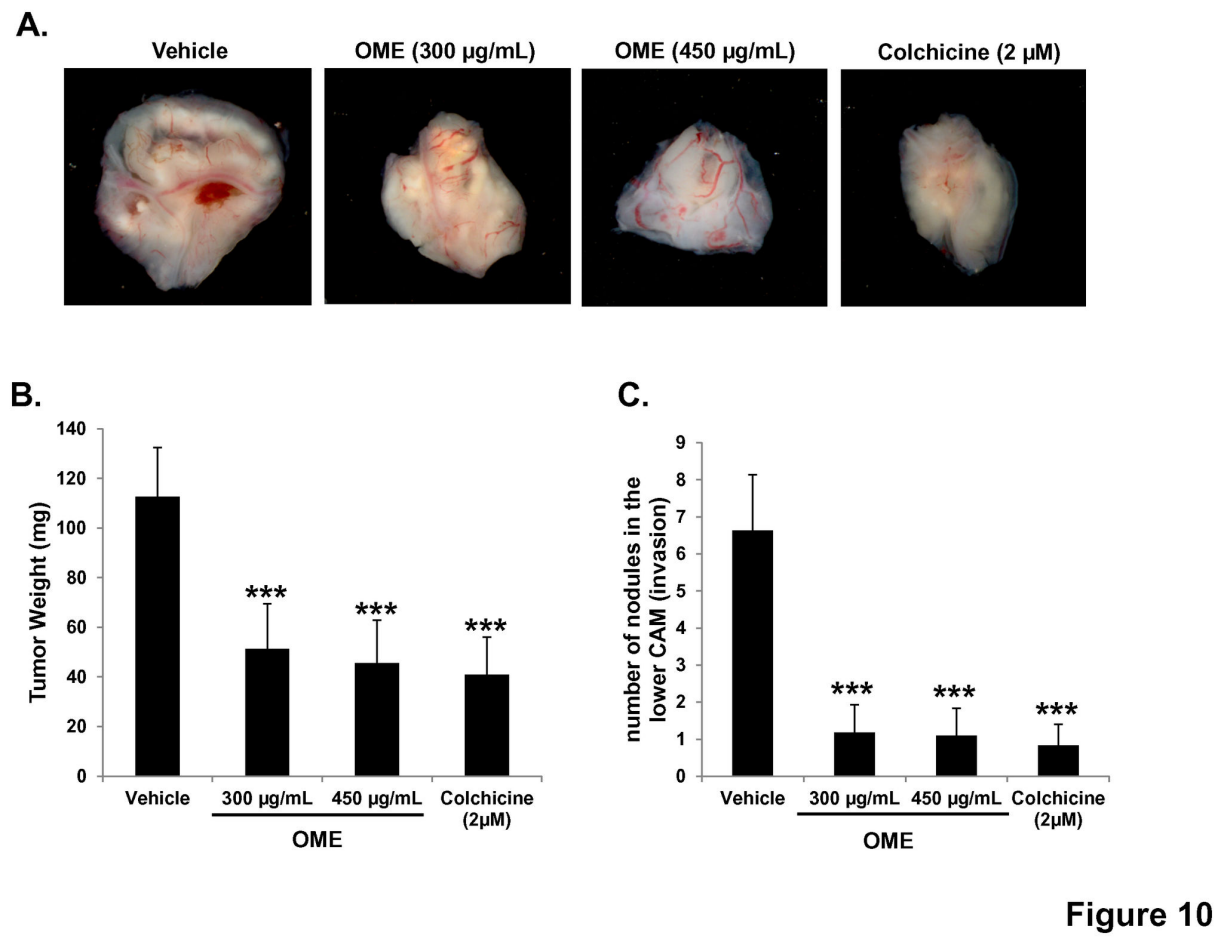
Anti-tumor growth and anti-metastatic activity of *O* *. majorana* on breast tumor in chick embryo chorioallantoic membrane model system. (A) MDA-MB-231 (1 x 10^6^) cells were grafted on the CAM of 10 day (E10) chick embryo. Tumors were treated every 48 h with OME as described in Materials and Methods. At E19, tumors were collected and weighted. (B) Quantification of tumor weight in vehicle, colchicine and indicated concentrations of OME-treated chick embryo. (C) Anti-metastatic effect of *O* *. majorana* . Quantification of nodules observed in the lower CAM of chick embryo treated with vehicle, colchicine or indicated concentrations of OME. Columns represents mean; bars represents SEM. *** significantly different at *p* < 0.0005.

To further confirm the *in vitro* anti-breast cancer activities of *O* *. majorana* , we decided to investigate its effect on tumor growth *in vivo* by using the chick embryo model. MDA-MB-231 cells were grafted on the chorioallantoic membrane (CAM) and formed tumors were treated every 48 h with vehicle, colchicine (2 µM) or increased concentrations of OME (300 and 450 µg/mL). At E 19, tumors were recovered from the upper CAM and weighted. As it is shown in Figure 10 A and B, OME significantly inhibited tumor growth compared with the vehicle treatment. In fact, concentrations of 300 and 450 µg/mL OME led to reduced tumor growth by 55 and 60% respectively. Similar effect (65% inhibition) was obtained with 2 µM colchicines. Toxicity was evaluated by comparing the number of dead embryos in OME-treated and control (vehicle- and colchicine-treated) embryos. We found that OME showed no toxicity to the embryo (data not shown).

We next assessed for the ability of OME to inhibit metastasis by counting the number of nodules in the lower CAM in vehicle, colchicines and OME treated tumors. An average of 6.6 nodules were counted in the lower CAM of vehicle-treated chick embryo, while an average of 1.1 nodules only were counted in 300 and 450 µg/mL OME-treated embryo (Figure 10C). All together, our data clearly demonstrates that OME could efficiently inhibit breast tumor growth and metastasis *in vivo*.

## Discussion

Tumor invasion and metastasis is a multistep process involving cell adhesion, proteolytic degradation and migration through the ECM and angiogenesis. Common cancer treatment drugs aim at blocking cell cycle progression, inducing cell death and/or inhibiting tumor migration and invasion. Cancer chemoprevention through these events has been reported for several natural compounds [15,17,42–44]. Nowadays, there is a growing interest in combination therapy using multiple anticancer drugs affecting several targets/pathways. Herein, we demonstrate for the first time that *O* *. majorana* , at non-cytotoxic concentrations, possesses potent anti-metastatic activities against the highly invasive triple negative breast cancer cell line, MDA-MB-231. In fact, *O* *. majorana* efficiently inhibited the migratory abilities and induced homotypic aggregation of MDA-MB-231 cells, associated with an upregulation of E-cadherin expression. We also showed that *O* *. majorana* not only decreased the adhesion of MDA-MB-231 to HUVECs as well as their transendothelial migration, but also inhibited the secretion of the pro-angiogenic factor VEGF from both endothelial and breast cancer cells. In addition we demonstrate that *O* *. majorana* suppressed the expression and the activities of MMP-2 and MMP-9 and downregulated the expression of uPAR and ICAM-1. *O* *. majorana* also blocked IκB-α/NFκB and reduced Nitric Oxide (NO) production, both signalings involved in cancer cell invasion. Moreover, we demonstrated that *O* *. majorana* significantly inhibited tumor growth and metastasis *in vivo* in chick tumor growth assay.

It is well known that disruption of cell-cell adhesion during cancer progression is the initial stage required for the acquisition of invasive properties. Decreased cell-cell adhesion in cancer cells is often characterized by diminished expression of E-cadherin. Indeed, E-cadherin, a calcium-dependent, cell adhesion molecule, is considered as a tumor suppressor in breast cancer [45]. A decrease in E-cadherin expression is a critical and necessary event required in the disruption of cell-cell adhesion and thus for the acquisition of invasive phenotype of various tumors including breast cancer. In fact, downregulation or loss of E-cadherin during cancer progression is associated with aggressive behavior of the tumor and poor prognosis [46]. Conversely, expression of E-cadherin led to a reduced progression and invasion of breast cancer cells [47]. In the present study, we demonstrated that *O* *. majorana* inhibited cell migration and promoted homotypic cell-cell aggregation and induced overexpression of E-cadherin in the MDA-MB-231 cells. We postulate that *O* *. majorana* exerts its anti-migratory effect on breast cancer cells, at least partly, through reactivation of the expression of E-cadherin gene. In fact we showed that OME was able to induce transactivation of E-cadherin promoter in transfected cells (Figure 3D). Induction of E-cadherin expression promotes then homotypic aggregation of OME-treated MDA-MB-231 cells. Thus, E-cadherin overexpression is one of possible mechanism by which *O* *. majorana* exerts its anti-invasive effect on the MDA-MB-231 cells. Recent studies showed that treatment with histone deacetylase inhibitors such as SAHA and trichostatin A strongly inhibited the migration and invasion of breast and prostate cancer cells and caused an upregulation of E-cadherin [48,49]. Histone deacetylases (HDAC) inhibitors, promising anticancer agents, have been shown to mediate their effects by activating the transcription of specific genes through histones hyperacetylation. Interestingly, we have recently shown that *O* *. majorana* induces hyperacetylation of histone H3 and H4 at non-cytotoxic concentrations [31]. Moreover, *O* *. majorana* has been shown to contain luteolin [50], a dietary flavonoid with histone deacetylase inhibitor activity, which was also shown to inhibit the invasive potential of MDA-MB-231 cells [33]. Based on these findings, we can suggest that one possible mechanism by which *O* *. majorana* exerts its anti-migratory and anti-metastatic effects on MDA-MB-231 involves an upregulation of E-cadherin gene expression through histone hyperacetylation. However, at this stage, we cannot rule out the involvement of other mechanism(s) involved in this regulation. Further investigation will be needed to decipher the exact mechanism by which *O* *. majorana* exert its effect on E-cadherin expression.

The dissemination of cancer cells from the primary tumor is another crucial event in the process of cancer invasion and metastasis, which involves the degradation of the ECM and the components of the basement membrane through proteases. Of these proteases, MMPs such as MMP-2, MMP-9 and the uPA are thought to play a key role in cancer cell invasion and metastasis [51,52]. It has been showed that increasing expression of MMPs in breast cancer cells correlates with increasing aggressiveness of breast cancer cell growth and metastatic potential [53]. Therefore, inhibiting the activity or the expression of these proteases can be considered as potential therapeutic targets against breast cancer. Interestingly, here we clearly demonstrate that *O* *. majorana* exerts its anti-invasive affect against MDA-MB-231 cells by significantly downregulating the expression of uPAR, MMP-2 and MMP-9 and decreasing the activity of these two proteases and consequently reducing ECM degradation.

The ability of tumor cells to metastasize also largely depends on their ability to adhere and transmigrate though endothelial cells. Cancer cell-endothelial cell interaction is mediated by various adhesive molecules such as intracellular adhesion molecule-1 (ICAM-1), vascular cell adhesion molecule-1 (VCAM-1) and E-selectin [54].

Studies have shown that the level of ICAM-1 protein expression on the cell surface positively correlated with metastatic potential of several breast cancer cell lines [8]. Moreover, downregulation of ICAM-1 at the protein and mRNA levels strongly inhibited human breast cancer cell invasion [8]. Tanshimone I, a natural compound derived from medicinal plant, *Salvia* *miltiorrhiza* , efficiently inhibited the adhesion of MDA-MB-231 cells to HUVECs by downregulation the expression of ICAM-1 and VCAM-1 [55]. Recent study revealed that ICAM-1 expression was associated with a more aggressive breast tumor phenotype [56]. Based on these findings, ICAM-1 represents a potential target in breast cancer treatment. Our study revealed that *O* *. majorana* was able to reduce the expression level of ICAM-1 protein in MDA-MB-231 cells and blocked their adhesion to the human vascular endothelial cells (HUVECs). Hence, we propose that downregulation of ICAM-1 expression could be one mechanism by which *O* *. majorana* blocks MDA-MB-231 cells capabilities to adhere to HUVECs and consequently preventing their metastasis.

Angiogenesis, a process by which new blood vessels forms, is crucial for tumor growth and metastasis. Blockade of angiogenesis can inhibit both tumor growth and metastasis [57]. Thus inhibition of angiogenesis can be considered as a promising strategy in cancer therapy [57,58]. One possible way to block angiogenesis is to target pro-angiogenic factors secreted by tumor cells. VEGF is the major pro-angiogenic protein expressed in 60% of breast cancer patients at the time of first diagnosis [59]. Interestingly, we found that *O* *. majorana* significantly reduced the production of VEGF in both HUVECs and MDA-MB-231 cells and thus suggesting that OME not only inhibit breast cancer cell invasion but could also block angiogenesis.

The NFκB signaling pathway is known to regulate the expression of various genes involved in the process of tumor metastasis. Elevated levels of NFκB are frequently detected in breast cancer cells. Studies showed that inhibition of NFκB activity could suppress metastasis in breast cancer cells. In fact, studies showed that inactivation of NFκB in MDA-MB-231 breast cancer cells inhibit the expression of many downstream target genes involved in tumor metastasis such as MMP-2 [53], MMP-9 [16,60,61], VEGF [61], ICAM-1 [16] and uPAR [16]. The phosphorylation of IκB, by the IKKβ subunit of the IKK serine kinase complex, is a crucial step in the activation of NFκB. In fact, phosphorylation of IκB triggers its polyubiquitination and proteasome-mediated degradation with consequent NFκB nuclear localization [62]. Nuclear NFκB can then upregulate the transcription of its target genes. Hence, one way to inactivate the constitutively activated NFκB signaling in cancer cells is to block the phosphorylation of Iκb by inactivating the IKK complex. Interestingly, in the present work, we demonstrated that *O* *. majorana* inhibited the phosphorylation of IκB and reduced the protein level of nuclear NFκB and thus suggesting that *O* *. majorana* might negatively regulate the activity of NFκB possibly by affecting the activity of the IKK complex. It is noteworthy to mention that *O* *. majorana* significantly reduced the expression of several NFκB downstream target genes (MMP-2, MMP-9, uPAR, ICAM-1 and VEGF) involved in tumor metastasis. It appears then, that the inhibitory effect on NFκB could account in the anti-metastatic effects of *O* *. majorana* .

Nitric Oxide (NO), synthesized by several nitric oxide synthases (NOSs), nNOS/NOS-1, iNOS/NOS2 and eNOS/NOS3 is a signaling molecule that regulates several physiological responses such as vasodilatation, cell migration, immune reactions and apoptosis [55]. Interestingly, various studies have shown that NO can both promote and inhibit tumor progression and metastasis. The pro- or anti-tumorigenic activities of NO have been related to the p53 status [41,63]. It has been shown that NO-mediated apoptosis in leukemia cells requires wild-type p53 [64]. On the other hand, it has been shown that iNOS/NOS2 expressing carcinoma cells with mutant p53 have accelerated tumor growth and increased VEGF production [65]. NO was shown to promote cancer progression through an activation of oncogenic signaling pathways including the extracellular signal-regulated kinases (ERK) 1/2, phosphoinositide 3-kinases (PI3K)/AKT, and cMyc [32]. Recent studies showed that increased iNOS/NOS2 and consequently NO production, predicted poor survival in women with estrogen receptor α–negative (ER-negative) breast tumors. Moreover, exposure to NO enhanced cell motility and invasion of Estrogen Receptor negative ER(-) cells [66]. It appears then that discovery of inhibitors targeting NO production may be particularly efficacious against ER(-), mutant p53 breast cancer patients. In the present study we showed that *O* *. majorana* efficiently reduced the level of NO production in the MDA-MB-231 in dose-dependent manner suggesting that *O* *. majorana* might exert its anti-metastatic effect, at least partly, by modulating the NO production in MDA-MB-231 cells. As such, NO production may be an important target for chemoprevention and therapy by *O* *. majorana* for the ER(-), mutant p53 MDA-MB-231 cells.

In summary, this study clearly demonstrated, for the first time, that *O* *. majorana* possess an anti-invasive and anti-metastatic effects against the highly proliferative and highly invasive human MDA-MB-231 breast cancer cell line by modulating the activity and/or the expression of proteins regulating the process of cellular migration, adhesion invasion and angiogenesis such as E-cadherin, ICAM-1, MMP-2, MMP-9, uPAR and VEGF at least partly through inhibition of the NFκB and NO signaling pathways. Our results also showed that *O* *. majorana* inhibited tumor growth and metastasis in an *in vivo* tumor growth assay. Thus, our current study along with our previous findings, identify *Origanum* *majorana* as a promising chemopreventive and therapeutic candidate that inhibits breast cancer growth and metastasis by modulating the expression and activities of several targets. Nowadays, there is a growing interest in combination therapy using multiple anticancer drugs affecting several targets/pathways, thus *O* *. majorana* certainly merits a lot of attention for further explorations to identify novel compounds for breast cancer.
